# Degradation Studies Realized on Natural Rubber and Plasticized Potato Starch Based Eco-Composites Obtained by Peroxide Cross-Linking

**DOI:** 10.3390/ijms19102862

**Published:** 2018-09-20

**Authors:** Elena Manaila, Maria Daniela Stelescu, Gabriela Craciun

**Affiliations:** 1 Electron Accelerators Laboratory, National Institute for Laser, Plasma and Radiation Physics, 409 Atomistilor Street, 077125 Magurele, Romania; elena.manaila@inflpr.ro; 2 National R&D Institute for Textile and Leather—Leather and Footwear Research Institute, 93 Ion Minulescu Street, 031215 Bucharest, Romania; dmstelescu@yahoo.com

**Keywords:** natural rubber, plasticized starch, polyfunctional monomers, physical and mechanical properties, cross-link density, water uptake

## Abstract

The obtaining and characterization of some environmental-friendly composites that are based on natural rubber and plasticized starch, as filler, are presented. These were obtained by peroxide cross-linking in the presence of a polyfunctional monomer used here as cross-linking co-agent, trimethylolpropane trimethacrylate. The influence of plasticized starch amount on the composites physical and mechanical characteristics, gel fraction and cross-link density, water uptake, structure and morphology before and after accelerated (thermal) degradation, and natural (for one year in temperate climate) ageing, was studied. Differences of two orders of magnitude between the degradation/aging methods were registered in the case of some mechanical characteristics, by increasing the plasticized starch amount. The cross-link density, water uptake and mass loss were also significant affected by the plasticized starch amount increasing and exposing for one year to natural ageing in temperate climate. Based on the results of Fourier Transform Infrared Spectroscopy (FTIR) and cross-link density measurements, reaction mechanisms attributed to degradation induced by accelerated and natural ageing were done. SEM micrographs have confirmed in addition that by incorporating a quantity of hydrophilic starch amount over 20 phr and by exposing the composites to natural ageing, and then degradability can be enhanced by comparing with thermal degradation.

## 1. Introduction

Natural rubber (NR) is renewable, non-toxic, has excellent physical properties and due to its low price is the most used elastomer worldwide in industry or in a variety of applications in which the final products are in contact with food or potable water [1]. The most common physical–chemical treatment of rubber is curing (cross-linking, vulcanization) by sulphur, peroxides, ultraviolet light, electron beam, and microwave irradiation, but sulphur and peroxide curing systems still remain the most desirable. The application of sulphur systems leads to the formation of sulphidic cross-links between elastomer chains [2]. In peroxide curing, high thermal stabile C–C bonds are formed. Therefore, peroxide cured elastomers exhibit high-temperature ageing resistance and low compression that is set at high temperatures. There are some disadvantages when compared peroxide to sulfur cured systems, such as low scarce safety and worse dynamic and elastic properties of vulcanizates [3]. But, the cross-linking with peroxides can be effectively improved by the use of co-agents [4,5,6], because they are able to boost peroxide efficiency by suppressing side reactions to a large extent, like chain scission and disproportionation [3,7], or by the formation of co-agents bridges between polymer chains as extra cross-links [8,9]. Trimethylolpropane trimethacrylate (TMPT), is a polyfunctional monomer that is used as co-agent [10,11] in order to improve the cross-linking process because is able to increase the rate state of cure and, as a consequence, to improve the physical properties of the processed material [8,12]. Anyway, the NR is used in form of mixtures, which generally contain active fillers, plasticizers, cross-linking agents, and other ingredients that give different characteristics to the final product [12,13]. New environmental-friendly elastomeric materials can be obtained by the use of natural fillers in NR and other rubber blends instead of hazardous active fillers, such as silica or carbon black, which are very well known for the harmful effects on human health [14]. So, it is preferable to replace the classics with other compatible types of filler that should maintain, or even improve, the mechanical and usable properties of NR or other rubber products. Because of the increased interests in replacement of non-renewable rubber materials with some based on components originated from natural resources, the use of natural fillers is considered as being a promising solution [15,16]. Even though their nature is known to be hydrophilic, starches are considered among the most promising available natural biopolymers and may successful candidate for the development of novel composite materials based on NR [17,18]. These are cheap, abundant available, biodegradable, recyclable, renewable, and present thermoplastic behavior [19,20,21]. Thermoplastic properties of starch are obtained by the disruption and plasticization of native starch with plasticizers agents (glycerol, water, and other polyols) [21,22,23].

The goal of the paper is to present the obtaining and characterization of a new environmental-friendly elastomeric composite that is based on NR and plasticized starch (PS). The cross-linking method was by the use of peroxide in the presence of the trimethylolpropane trimethacrylate (TMPT). The elastomeric composite behavior under accelerated (thermal) aging and natural aging in temperate climate for one year was investigated, because it is very well known that starch-based plastics have some drawbacks, including limited long term stability caused by water absorption, ageing caused degradation, poor mechanical properties and bad processability. Also, the influence of PS amount on the physical and mechanical properties, cross-linking density rate and behavior in aqueous environment, before and after aging was studied. The novelty of the present study consists in the replacing of conventional fillers (silica, carbon black) with an environmental friendly filler (plasticized starch) in order to obtain composites having improved cross-linking density and mechanical properties, and over these, a high degradation degree in natural environment.

## 2. Results and Discussion

### 2.1. Physical and Chemical Characteristics

**Figure 1:**
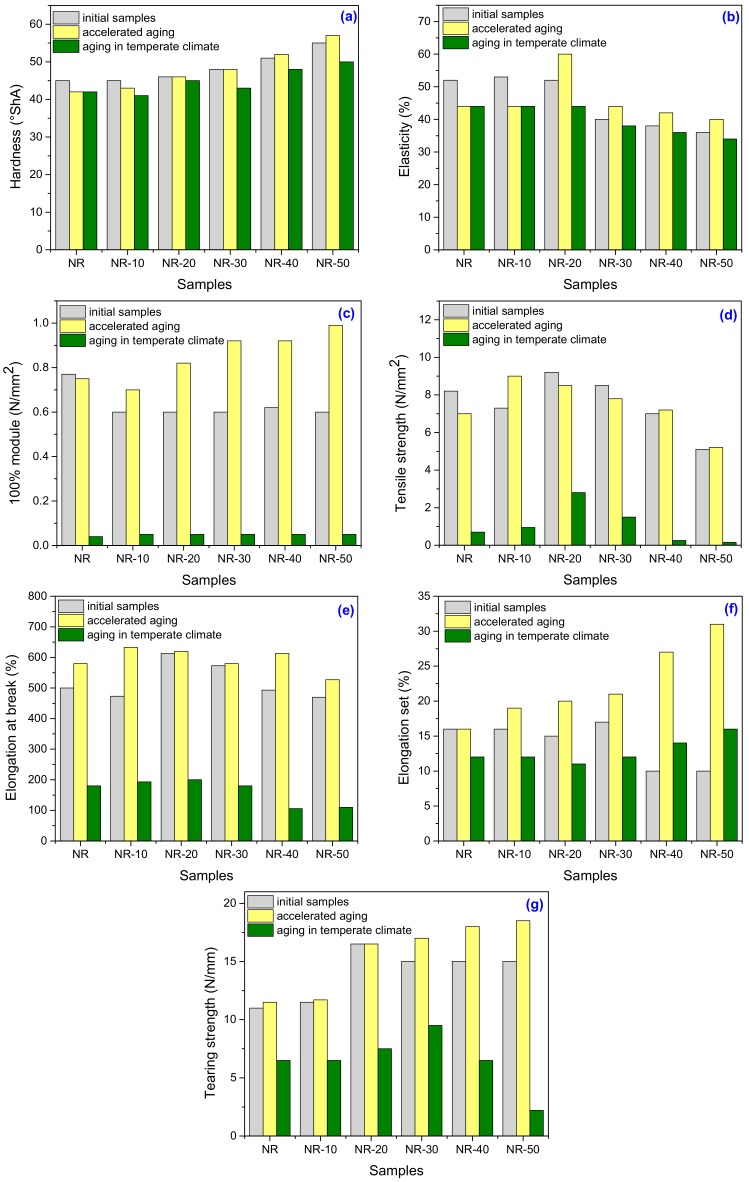
The Hardness (**a**), Elasticity (**b**), 100% Modulus (**c**), Tensile strength (**d**), Elongation at break (**e**), Elongation set, and (**f**) Tearing strength (**g**) variations as a function of PS amount and aging method.

The results of mechanical tests that have been made on unfilled NR and NR filled with PS composites (NR–PS), before and after aging are summarized in Figure 1. An improvement of mechanical properties before aging, excepting 100% Modulus (Figure 1c), due to the plasticized starch introduction, can be observed.

#### 2.1.1. Mechanical Properties of Unfilled and Filled NR before Aging

As it can be seen from Figure 1a, hardness has slowly increased with the PS amount in blend increasing. This is due the reinforcement effect of PS, which incorporated in the NR matrix has conducted to a reduction of plasticity and flexibility of rubber chains and the composite become more rigid [14,24]. Irrespective of the PS amount added, the increase in hardness did not exceed 12.5% for unfilled NR. The results that are presented in Figure 1b show that the unfilled NR has exhibited a higher elastic response when compared to that of NR-PS samples. The composite having 10 phr of PS has a better elastic behavior than unfilled NR. The higher dynamic stiffness of the samples containing over 20 phr of PS can be the reason of the elasticity decreases [25]. The same results were obtained for 100% Modulus Figure 1c. The results presented in Figure 1d show that the tensile strength, which strongly depends on effective and uniform stress distribution, increases as the amount of PS increase, up to 20 phr. As the PS amount still increases, the tensile strength started to decrease by about 45%, because the PS tend to agglomerate, leading to insufficient wetting of filler and bad interface with the matrix. So, the PS acts as flows limiting the tensile strength. Also, another reason may be connected with the presence of some voids trapped in the composite during processing, so a specific attention has to be accorded to the preparation technique when is dealing with high filler loading in order to assure its well dispersion [26,27].

As it can be seen from Figure 1e, elongation at break has the same behavior as tensile strength on the entire PS concentration range. The decrease highlighted over 20 phr of PS, is the consequence of the appeared restriction in the movement of molecular chains, which has lead on a negative effect upon the sample ductility [14,28,29]. Figure 1f shows that the elongation set has decreased with the PS amount in blend increasing, a fact that indicates also an increase in cross-link density. The decrease in residual elongation shows that the sample is vulcanized and thus returns to its original shape easily [14,24]. Figure 1g shows also the same variation trend of tearing strength as tensile strength and elongation at break. So, we can conclude that the PS has a reinforcing effect on NR when it is loaded up to 20 phr.

#### 2.1.2. Mechanical Properties of Unfilled and Filled NR after Aging

##### Mechanical Properties of Unfilled and Filled NR after Accelerated (Thermal) Ageing

A specimen from each sample was subjected to thermal ageing in an air-circulating oven at 70 °C for 168 h, in order to evaluate the rubber compound properties before and after aging. As it can be seen from Figure 1a–g and Table 1, after the thermal aging, mechanical properties are modified for all the tested composites.

The resistance of rubber based composites to thermal aging is considered as being an essential requirement for better service performance. The increasing of elasticity, 100% modulus, tensile strength, elongation at break, tensile set, and tearing strength of NR-PS composites is due to the presence of peroxide free radicals that were not involved in the cross-linking reactions and that lead to the formation of few new cross-links during thermal aging. The phenomenon is well known as post-curing during aging [30,31]. On the other hand, elasticity, 100% modulus and tensile strength of the unfilled NR have diminished after thermal aging due to the post-curing during aging by which excessive cross-links were formed [30,32]. It seems that the PS presence, even in the small amount of 10 phr, has delayed the formation of excessive cross-links in NR-PS as compared with the unfilled NR.

##### Mechanical Properties of Unfilled and Filled NR after 1 Year of Natural Ageing in Temperate Climate

Another specimen from each sample was subjected to natural ageing for one year in temperate climate between March 2017 and March 2018, in Bucharest, Romania. In order to determine the effect of outdoor exposure, samples were suspended in vertical position on a special dryer. The influence of the natural environment (heat, cold, frost, sunlight, oxygen, moisture, precipitations) upon the degradation was evaluated by comparing the samples mechanical properties before and after aging processes.

From the results presented in Figure 1a–g and Table 2, it can be seen the important changes suffered by all mechanical properties of unfilled and filled NR composites, after one year of natural ageing in temperate climate staying. The negative modifications of mechanical properties clearly show the sample degradation during the natural process. So, 100% modulus and tensile strength present an important falling-off that indicates structural changes in rubber chains due to the cross-links dissociation.

As a consequence, the composites lose the elastic properties and the ability to act as an effective matrix material to transmit stress [30,33]. An important decrease of elongation at break was also observed. This is as a consequence of both cross-linking and scission reactions, which negative modify the elastic nature of rubber chains [30,34,35].

#### 2.1.3. Gel Fraction and Cross-Link Density

**Figure 2:**
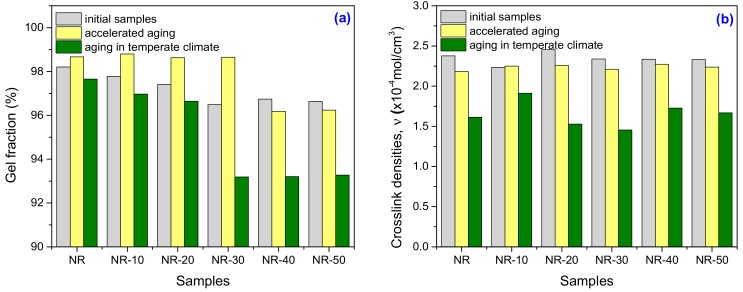
Gel Fraction (**a**) and Cross-link density (**b**) variations as a function of plasticized starch (PS) amount and aging method.

Figure 2a,b presents the results regarding the gel fraction and cross-link density investigations before and after aging. In Table 3, the percentage changes of these parameters for unfilled and filled NR after one year of natural ageing in temperate climate are presented.

As it can be seen from Figure 2a,b, before being subjected to aging tests, the introduction of PS has induced a decrease of samples gel fraction and a not so significant modification of cross-link density. Continuing the loading with PS over 20 phr, the cross-link density remains on a path. The slight variations of cross-link density may be explained by the changing in phase structure character of NR in which the PS was introduced. But, for most applications, the cross-link density should not be so high and it must be sufficient to give the rubber mechanical integrity so that it can bear loads and present deformation recovery. A high cross-link density immobilized the polymer chains, fact that lead to a hard and brittle rubber [36,37].

Figure 2a,b and Table 3 show that after the accelerated (thermal) aging, an increase in the gel fraction is observed for both unfilled and filled NR composites up to the loading with 30 phr. Over this, the gel fraction has decreases and remains on a path. The cross-link density was more affected by the thermal aging. But, after one year of natural degradation in temperate climate, all the samples gel fractions and cross-link densities have strongly decreased. NR samples have showed a reduction of 32.13%, while NR-PS samples of 37.81% for the loading of 20 phr and 37.83% for 30 phr, respectively. The results are in agreement with those that were obtained in mechanical tests and prove a clear degradation of the composites.

### 2.2. Structural and Morphological Characteristics

#### 2.2.1. Spectral Characterization by Fourier Transform Infrared Spectroscopy Analysis

**Figure 3:**
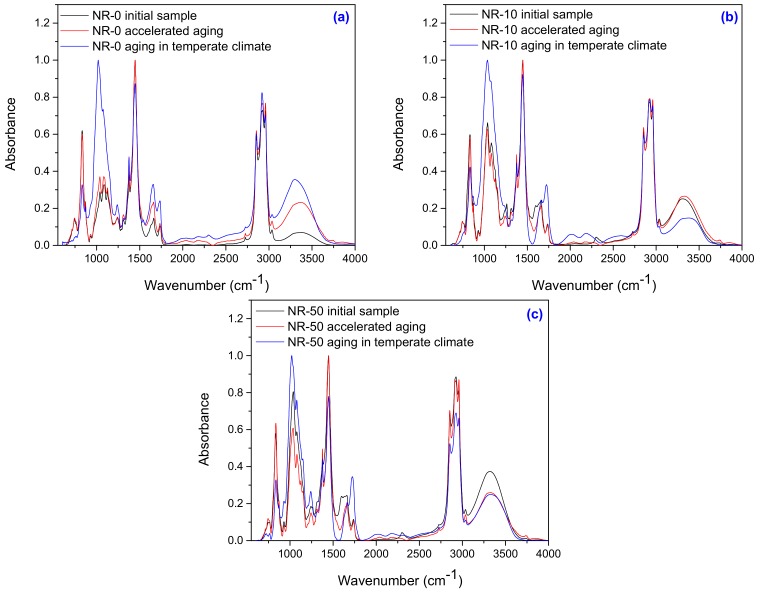
Infrared spectra in the range of 600–4000 cm^−1^ for samples unfilled (**a**), filled with 10 phr PS (**b**) and with 50 phr PS (**c**).

In order to investigate structural modification of filled NR as compared with unfilled NR before and after aging, the spectral characterization by Fourier Transform Infrared Spectroscopy (FTIR) in the range of 600–4000 cm^−1^, was done. The results are presented in Figure 3a–c.

The assignments of the main bands in the NR and NR-PS before and after aging are summarized in Table 4. A comparative analysis between the FTIR spectra of NR (Figure 3a) and NR-PS (Figure 3b,c) highlights notable differences (Table 4).

Thus, the band that appeared at 1081 cm^−1^ (C–O–C) indicates the grafting of PS on NR [38]. Also, the band at 930–925 cm^−1^ can be attributed to the skeletal mode vibrations of α-(1-4) glycosidic linkage (C–O–C) and the one between 1100 cm^−1^ and 1030 cm^−1^ is characteristic of the anhydrous glucose ring C–O stretch [39,40]. The absorption bands at 3380 cm^−1^ that appear in NR-0 before aging treatment (Figure 3a) were identified to the proteins from NR [41]. After degradation, the spectra indicate that the intensity of the broad bend near 3380 cm^−1^ has increases. The broad band at 3380 cm^−1^ may be due formation of hydroxyl group (–OH) as a result of the degradation by oxidation [38,42]. It can be seen that after 1 year of natural ageing in temperate climate, all these bands have increased in intensity. The decreasing of *cis*-1,4 double bonds number in the polyisoprene chain at 833 cm^−1^, the formation of hydroxyl group at 3380 cm^−1^, the appearance of ketone and aldehyde groups between 1736–1722 cm^−1^, and the increasing of glycosidic linkage at 930–925 cm^−1^ may be interpreted as consequences of the degradation induced by the accelerated and natural aging processes to which samples have been subjected. It can be seen that after natural ageing, all these bands have increased in intensity. All of these, correlated with the decreasing of *cis*-1,4 double bonds number in the polyisoprene chain at 870–830 cm^−1^, the formation of hydroxyl group at 3380 cm^−1^, the appearance of ketone and aldehyde groups between 1736–1722 cm^−1^ and the increasing of glycosidic linkage at 930–925 cm^−1^ may be interpreted as consequences of the degradation. Also, the formation of carbonyl or hydroxyl bonds (>C=O and –OH) and carboxylate or conjugated ketone (RCOOH and R_2_C=O) is demonstrated by the occurrence of the absorption bands between 1260–1400 cm^−1^ and 1550–1690 cm^−1^, respectively [43]. The cross-linking degree and molecular masses decreasing are due to the cleavage of the macromolecules, as demonstrated by the appearance of the absorption bands at 1370–1380 cm^−1^, 2850–2880 cm^−1^, and 2950–2980 cm^−1^ that correspond to –CH_3_ groups. The slight increasing of the absorption bands at 1640–1660 cm^−1^ and 800–900 cm^−1^ due to the number of double bonds increasing, as well as the modification of absorption bands at 800–900 cm^−1^, 1650–1680 cm^−1^, and 3010–3040 cm^−1^ that correspond to the changes in the degree of substitution of carbon atoms of the double bond are connected with the degradation process [43].

#### 2.2.2. Mechanisms of Natural Degradation Reactions for NR and NR-PS

In temperate climate, the main ageing processes in NR are the oxidative and thermal-oxidative degradation (photo-degradation) and UV/ozone degradation [36,44].

The initial step of oxidative and thermal-oxidative degradation consists in free-radicals formation on the NR chain by hydrogen abstraction.

**Figure 4:**
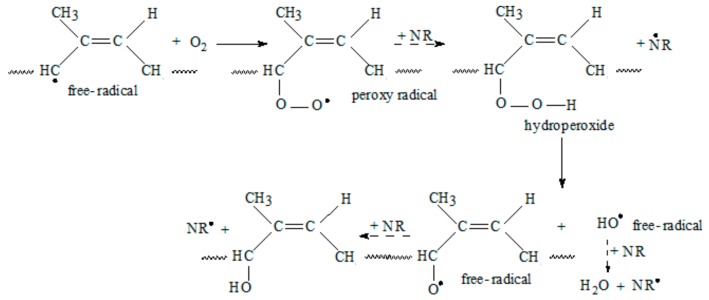
Propagation step of oxidative degradation.

The propagation of oxidative degradation takes place in several stages as it can be seen from Figure 4. The propagation first step is the reaction of a free radical with an oxygen molecule (O_2_) to form a peroxy radical (NR–O–O•), which then abstracts a hydrogen atom from another polymer chain to form a hydroperoxide (NR–O–O–H). The hydroperoxide splits then into two new free radicals, (NR–O• and •OH) that abstract another hydrogen and from other polymer chains.

**Figure 5:**
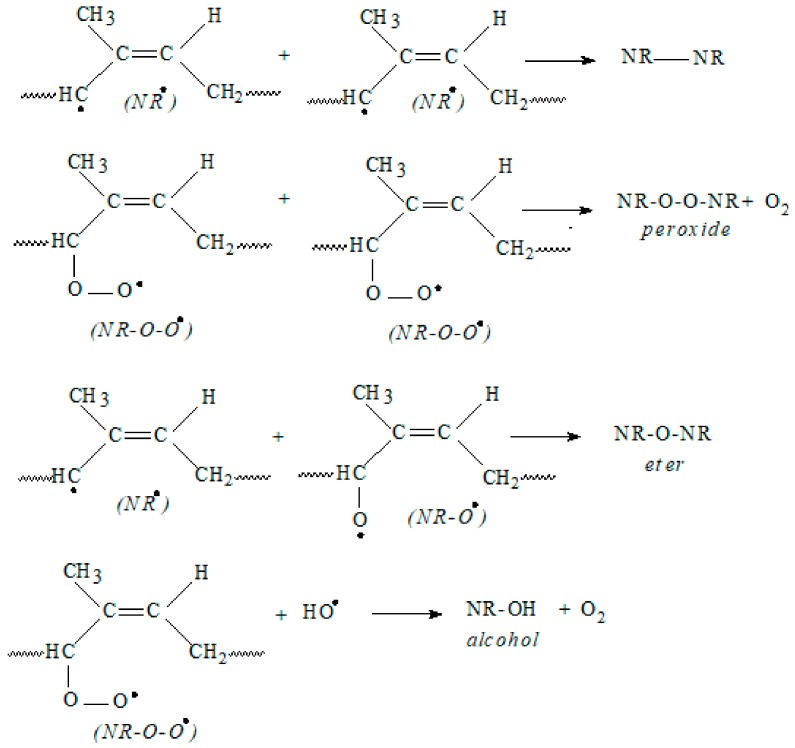
Termination of oxidative degradation by free radical recombination.

The termination of the reaction is achieved by the recombination of two radicals or by disproportionation/hydrogen abstraction, as it can be seen from Figure 5.

**Figure 6:**
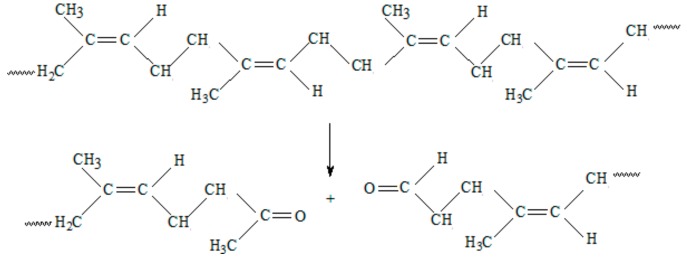
Termination of oxidative degradation by chain scission.

The results of these reactions are the polymer enbrittlement and cracking. On the other hand, termination by chain scission, as presented in Figure 6, results in the decrease of the molecular weight leading to softening of the polymer and reduction of the mechanical properties [44].

**Figure 7:**
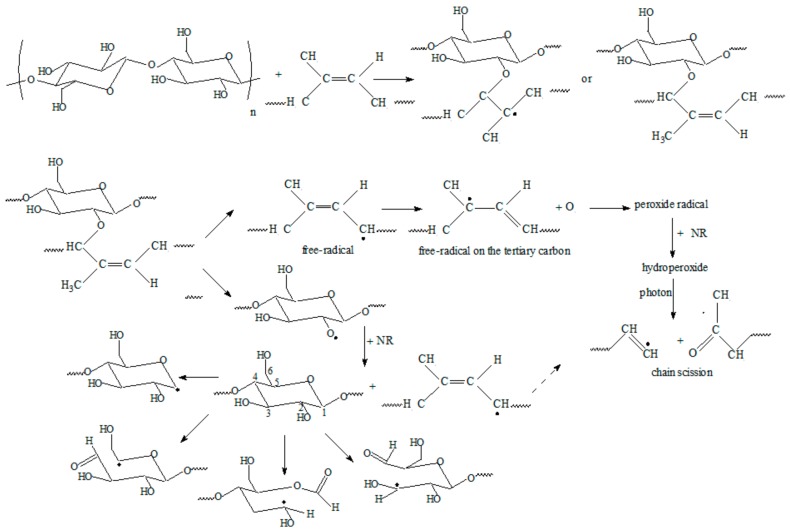
The photo-oxidation reaction on NR—starch bond.

In photo-degradation, if free radicals are directly produced by UV radiation, then all of the subsequent reactions are similar to those of thermal-oxidative degradation, including chain scission, cross-linking, and secondary oxidation. Photo-oxidation can also occur by breaking the bonds that are created between NR and PS (Figure 7). Starch may form other radicals by the cleavage of a glycosidic bond and from β-fragmentation of an oxygen-centred radical resulting from cleavage of a glycosidic bond [45].

**Figure 8:**
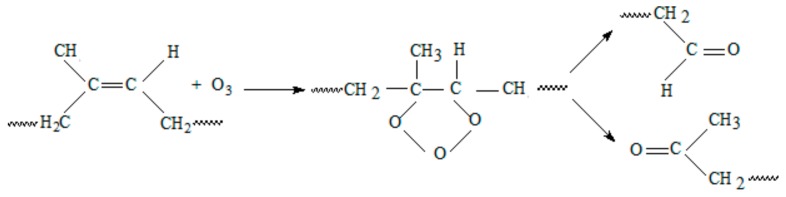
The ozone attack on NR chain.

Sunlight and ozone rapidly attack the unprotected polymers and can significantly reduce them service life [44]. Ozone, being more corrosive then the molecular oxygen will attack the polymer directly at the carbon-carbon double bond. As it can be seen from Figure 8, the ozone molecule attaches itself to the double bond creating a C_2_O_3_ ring. The chain scission results in products containing carbonyl groups.

Ozone degradation of vulcanized NR exhibits a very specific cracking, through which it is formed a hard surface layer [46]. The reaction with ozone, leads to chain scission of the polymer chain and the formation of polymeric peroxides that can also increase the rate of oxidative aging [36,47]. As those processes are related to substantial modifications of the macromolecular backbone, substantial damage in mechanical properties are expected, even at low rate of conversion (less than 0.1%) [36,37]. This was observed for all samples aged for one year in temperate climates, for both mechanical properties and cross-linking density.

#### 2.2.3. SEM Analysis

**Figure 9:**
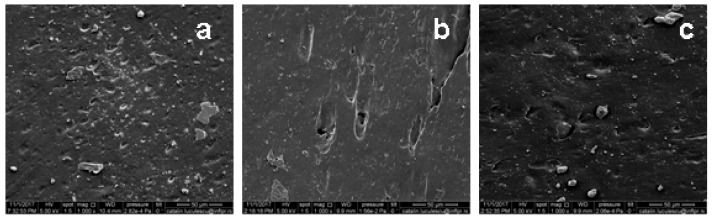
SEM micrographs of (**a**) NR, (**b**) NR-10, and (**c**) NR-50 samples, before one year of natural ageing in temperate climate, at magnification of 1000.

**Figure 10:**
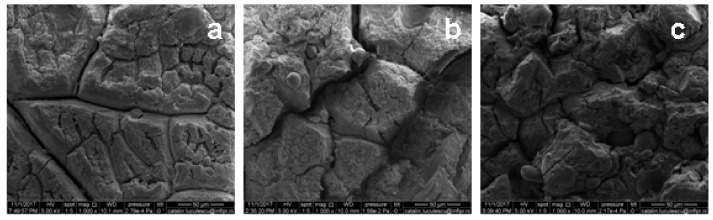
SEM micrographs of (**a**) NR, (**b**) NR-10, and (**c**) NR-50 samples, after one year of natural ageing in temperate climate, at magnification of 1000.

The morphological changes of unfilled and filled NR samples before and after one year of natural ageing in temperate climate were evaluated by surfaces SEM analysis. Before and after ageing, samples were immersed in toluene, in order to remove any split fragments or un-reacted materials. The results are presented in Figure 9 and Figure 10.

From Figure 9, it can be seen that, before aging, the NR and NR-PS samples surface looks smooth presenting only with small imperfections that can be caused by the presence of some impurities remained even after the immersion in toluene.

From Figure 10, the NR and NR-PS samples degradation after 1 year of natural ageing in temperate climate can be observed. The surfaces of all samples are rough, extensive surface cracks are formed, leading the appearance similar to a mosaic pattern. As the PS amount has increased, the roughness of surface sample also increased and more micro cracks are formed. The surface roughness can be attributed to the decreasing of elasticity, 100% modulus and tensile strength, as the filler loading was increased, but also to the dissociation of existing cross-links or to the structural changes in natural rubber chains [48]. In addition to the oxidative and ozone degradation, the outdoor thermal stress and the dust presence must be taken into account for the contribution to the cracks formation on the composite surface [48,49,50].

### 2.3. Water Uptake and Mass Loss

**Figure 11:**
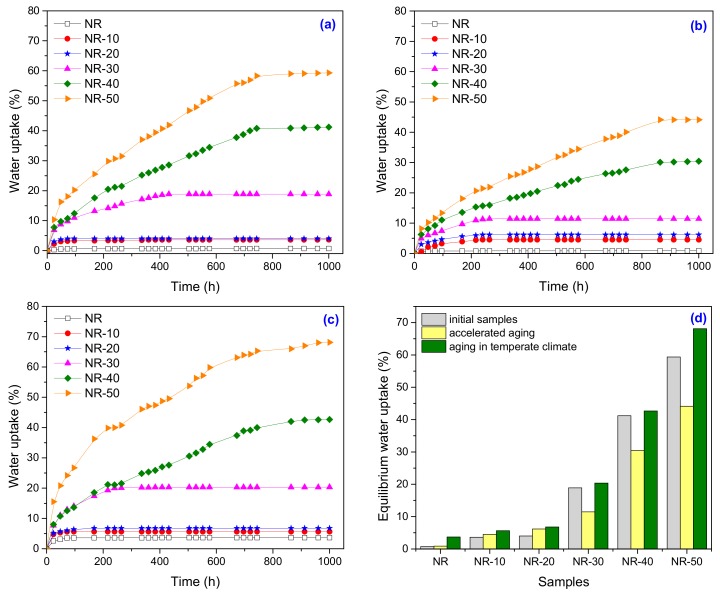
Water uptake (**a**) before aging, (**b**) after the accelerated ageing, (**c**) after one year of natural ageing in temperate climate, and (**d**) at equilibrium before and after ageing.

Water uptake tests were done before and after ageing in order to demonstrate the contribution of the strongly hydrophilic polar groups that appeared in the FTIR spectra of the composite, to the natural degradation. The results are presented in Figure 11a–d. As it can be seen, the water absorption of NR-PS composites is strongly dependent on the PS amount, increasing with the PS content due to the hydrophilic nature of starch and the greater interfacial area between the starch and the NR matrix.

Before ageing, the smallest absorptions were obtained for PS loadings up to 20 phr. Over this, the water uptake percentages were of 41.19% and 59.31% for 40 phr and 50 phr, respectively (Figure 11a). The post-curing phenomenon, after the thermal aging, has lead to the decrease of water uptake (Figure 11b). When the composites have been exposed to high temperature for longer period (7 days at 70 °C), the oxygen molecules from the air have been diffused into the surface, but some of them were immediately consumed by oxidation reactions to produce cross-linking, scission of the rubber chains or cross-links, and combination with molecular chains of rubber [51]. So, we can conclude that the water uptake is related with the cross-link densities of the composites. After 1 year of natural ageing in temperate climate, the water uptake percentages are increased, as it can be seen from Figure 11c,d. The phenomenon theory is talking about the difficulty of solvent molecule to penetrate the carbon linkages (C–C) because of the strong bonding and high rigidity [51,52]. But, in our study, the results show that the water molecules have easy penetrated the NR, the water uptake before and after one year of natural ageing in temperate climate being of 0.71% and 3.68%, respectively. In the same, the NR-PS composites have presented a great increase in water uptake percentages as comparing with the samples before and after thermal ageing, this indicating the decreasing of cross-link density and increasing of chain scissions. The disruption of C–C bonds in NR leads mainly to chain scission, while the disruption of C–O bonds from NR-PS causes the scission of grafting bonds [53]. Since the C–C and C–O bonding energies are comparable (346 vs. 358 kJ = mol), both phenomena can equally occur in degradation. Also, the appearance of some changes in the composites structure supported by FTIR results (decreasing of *cis*-1,4 double bonds number in the polyisoprene chain, the formation of hydroxyl group, the appearance of ketone and aldehyde groups and the increasing of glycosidic linkage) that can be interpreted as being consequences of the natural degradation, can also explain the increases in water absorption. From Figure 11d, clearly the results demonstrate the superiority of the natural ageing over thermal ageing. Also, it can be seen that high PS loadings create conditions for stronger degradation. Before and after immersion in water, all of the samples were weighed in order to determine the mass loss. The results are presented in Table 5.

From Table 5 it can be seen that NR does not presents significant mass losses before and after accelerated and natural ageing, higher being however after one year in temperate climate (0.026%). The filled NR has shows small mass differences, but increased with the PS amount, before and after ageing, the highest being of 2.99% for the case of NR-50 aged for one year in temperate climate. It should be noted that the mass losses for all of the samples loaded with PS over 30%, were smaller after thermal ageing than before. These results are the consequence of the post-curing effect and are in perfect agreement with those obtained after the mechanical properties evaluation. The results presented in Table 5 for NR and NR-PS after one year of natural ageing in temperate climate, show that higher PS content increases mass loss and enhances the degradation kinetics due to the starch hydrophilic nature, which leads to the moisture retaining. The higher is the starch content in the polymer, the higher is the moisture content that renders faster degradation. This can be correlated with the polymer sample’s gross morphology, which was observed to be physically changed, the surface being roughened over the degradation period as it can be seen in SEM micrographs from Figure 10 [54].

## 3. Materials and Methods

### 3.1. Materials and Samples Preparation

The raw materials that were used in the experiments were as follows: (a) Natural rubber (NR) for pharmaceutical use, Crep from Sangtvon Rubber Ltd. (Nakhon Si Thammarat, Thailand), in the form of white rubber sheets (Mooney viscosity of 67.64 ML1+4 at 100 °C, volatile materials content of 0.5%, nitrogen content of 0.45%, percentage of ash of 0.25%, impurities content of 0.026%); (b) Soluble potato starch produced by Lach-Ner Ltd. (Neratovice, Czech Republic), water insoluble substances 0.28%; loss on drying 16.9%, easily biodegradable: BOD_5_ −0.6 g/g and COD −1.2 mg/g); (c) Glycerine from SC Chimreactiv SRL (Bucharest, Romania) (free acidity 0.02%, density 1.26 g/cm^3^, purity 99.5%); (d) IPPD antioxidant (4010 NA) *N*-isopropyl-*N*-phenyl-phenylene diamine from Dalian Richon Chem Co. Ltd. (Dalian, China), 98% purity, molecular mass: 493.6374 g/mol; (e) Peroxide Perkadox, 40 dibenzoyl peroxide, from AkzoNobel Chemicals (Deventer, The Netherlands) (density 160 g/cm^3^, 3.8% active oxygen content, 40% peroxide content, pH 7); and, (f) TMPT DL 75 Luvomaxx, trimethylolpropane trimethacrylate as polyfunctional monomer from Lehmann&Voss&Co (Hamburg, Germany) (22% ash, pH 9.2, density 1.36 g/cm^3^, 75 ± 3% active ingredient).

Composites that are based on NR and PS have been obtained according with the recipes that are presented in Table 6. Mixtures were cross-linked with peroxide in the presence of TMPT; a polyfunctional monomer was used here as curing co-agent.

PS was obtained by mixing at 70 °C, starch (50%), water (20%), and glycerine (30%) for 15 min at 50–100 rpm until the homogeneity was attended. After, the homogeneous mixture has been left to rest for 1 h, then being introduced in the oven at 80 °C for 22 h and at 110 °C for another 2 h. Finally, has been left to cool down for at least 16 h in a dry place.

The blends were prepared on an electrically roller mixer. The constituents were added in the following sequences and amounts: NR that has been mixed in the roller mixer for 2 min, PS and glycerine (mixing time between 5 and 30 min), antioxidant (mixing time: 1 min), peroxide, and TMPT (mixing time: 1 min). After all of the ingredients were added, they were homogenized for another 2 min and then removed from the roller mixer in the form of a sheet. The sheets have been cured using moulds and vulcanization press in order to obtain rubber plates with the sizes of 150 × 150 × 2 mm^3^ being required for die punching test specimens. The compression temperature in the moulding machine was kept constant at 160 °C, for 20 min at a pressure of 300 kN. Cooling time was 10 min at 25 °C and 300 kN.

One specimen from each composite that have been obtained as above was subjected to accelerated ageing (thermal ageing) in an air-circulating oven at 70 °C for 168 h and another one to natural ageing for one year in temperate climate. Physical and mechanical properties, cross-linking density rate, behavior in aqueous environment, and structural and morphological investigations were done before and after ageing.

### 3.2. Laboratory Tests

#### 3.2.1. Mechanical Characteristics Determining

Hardness, elasticity, 100% Modulus, tensile strength, elongation, and tearing strength were measured. Hardness was measured according to ISO 7619-1/2011 on 6 mm thick samples, while using a hardness tester. Elasticity (the rebound resilience) was evaluated according to ISO 4662/2009 also on 6 mm thick samples, while using the Schob test machine. Tensile strength and tearing strength tests were carried out with a Schopper strength tester at testing speed of 460 mm/min, using dumbbell shaped specimens according to ISO 37/2012, and angular test pieces (Type II) according to EN 12771/2003, respectively.

#### 3.2.2. Sol-Gel Analysis

The sol-gel analysis, were performed on the cross-linked composites in order to determine the mass fraction of insoluble NR resulting from the network-forming cross-linking process. Samples having known mass were swollen in toluene for 72 h in order to remove any split fragments and un-reacted materials, and they were dried in air for six days and then in a laboratory oven at 80 °C for 12 h. Finally, samples were re-weighed and the gel fraction was calculated, as follows:

(1) $$Gel_{fraction}=\frac{m_{s}}{m_{i}}\times 100$$

where $m_{s}$ and $m_{i}$ are the mass of the dried sample after extraction and the initial mass of the sample, respectively [55,56].

#### 3.2.3. Cross-Link Density Determining

The samples cross-link density was determined on the basis of equilibrium solvent-swelling measurements in toluene at 23–25 °C, by application of the modified Flory-Rehner equation for tetra functional networks. Samples having thicknesses of 2 mm were initially weighed (*m_i_*) and immersed in toluene for 72 h. The swollen samples were dried to remove the solvent excess and weighed (*m_g_*) being covered, in order to avoid toluene evaporation during weighing. Traces of solvent and other small molecules were eliminated by drying in air for six days and then in an oven at 80 °C for 12 h. Finally, the samples were weighed for the last time (*m_s_*), and volume fractions of polymer in the samples at equilibrium swelling *ν*_2*m*_ were determined from swelling ratio *G,* as follows:

(2) $$\nu_{2m}=\frac{1}{1+G}$$

(3) $$G=\frac{m_{g}-m_{s}}{m_{s}}\times \frac{\rho_{e}}{\rho_{s}}$$

where, $\rho_{e}$ and $\rho_{s}$ are the densities of samples and solvent (0.866 g/cm^3^ for toluene), respectively.

Densities were determined by hydrostatic weighing method, according to SR ISO 2781/2010. The cross-linking densities *ν*, were determined from measurements in a solvent, while using the Flory–Rehner relationship:

(4) $$\nu =-\frac{Ln(1-\nu_{2m})+\nu_{2m}+\chi_{12}\nu_{2m}^{2}}{V_{1}\left(\nu_{2m}^{1/3}-\frac{\nu_{2m}}{2}\right)}$$

where, $V_{1}$ is the molar volume of solvent (106.5 cm^3^/mol for toluene), $\nu_{2m}$ is the volume fraction of polymer in the sample at the equilibrium swelling, and $\chi_{12}$ is the Flory-Huggins polymer-solvent interaction term (the value of and $\chi_{12}$ is 0.393 for toluene) [55,56].

#### 3.2.4. Structural and Morphological Measurements

Structural changes of NR and NR-PS before and after ageing, were highlighted by FTIR measurements that have been done while using the TENSOR 27 (Bruker, Bremen, Germany) FTIR spectrophotometer by ATR measurement method. The spectra were obtained from 30 scans mediation, realized in absorption in the range of 4000–600 cm^−1^, with a resolution of 4 cm^−1^.

Morphological measurements of NR and NR-PS, also before and after ageing, were done on the samples surfaces while using the scanning electron microscope FEI/Phillips (FEI Company, Hillsboro, OR, USA). For this, the samples were placed on an aluminum mount, sputtered with gold palladium, and then scanned at an accelerating voltage of 30 kV.

#### 3.2.5. Mechanisms of Degradation Reactions

Based on the results that were obtained by FTIR measurements, reaction mechanisms attributed to degradation induced by natural ageing were done. Generally, degradation can be induced by heat (thermal degradation), oxygen (oxidative and thermal-oxidative degradation), light (photo-degradation), and weathering (generally UV/ozone degradation) [44,45].

#### 3.2.6. Water Uptake Tests

The water uptake tests were done in accordance with SR EN ISO 20344/2004 in order to study the water absorption on NR and NR-PS before and after ageing. For this, the samples were dried in a laboratory oven at 80 °C for 3 h and then were cooled at room temperature in desiccators before weighing. Water absorption tests were conducted by immersing the samples in distilled water in beaker and then maintaining at room temperature (25 ± 2 °C). After immersion, the samples were taken out from the water at periodic intervals and the wet surfaces were quickly dried while using a clean dry cloth or tissue paper before weighing. Absorption was calculated from the weight difference. The percentage of samples weight gaining was measured at different intervals of time. The water uptake was calculated, as follows:

(5) $$\mathrm{Water}\;\mathrm{uptake}\;(\text{\%})=\frac{m_{t}-m_{i}}{m_{i}}\times 100$$

where, *m_t_* is the weight of the sample immersed in water at time *t* and *m_i_* is the initial weight of the oven-dried specimen.

Note: For mechanical, sol-gel analysis, cross-link determining, rubber-filler interaction, and water uptake tests before and after ageing, five samples were taken in work and the results are the averages of these five measurements.

## 4. Conclusions

A new elastomeric composite that is based on natural rubber and plasticized potato starch was obtained by the peroxide cross-linking method in the presence of the cross-linking co-agent trimethylolpropane trimethacrylate. The composite behavior before and after accelerated (thermal) aging and natural degradation in temperate climate for 1 year was investigated in terms of physical and mechanical characteristics. So, an improvement of mechanical properties, excepting 100% Modulus, due to the plasticized starch introduction, was observed before ageing. Also, by the increasing of the plasticized starch amount in the composite, excepting hardness, all other mechanical characteristics have started to decrease around the loading of 20 phr. Thus, we can say that the plasticized starch loading up to 20 phr has a reinforcing effect on natural rubber. By comparing the mechanical properties of the samples after ageing, we have observed the appearance of the post-curing during aging effect, reflected in minor modifications after the accelerated thermal ageing and notable negative modifications due to the natural process. These results are in accordance with those that were obtained by studying the gel fraction and cross-link density. Structural investigations through the FTIR technique before and after thermal and natural ageing were done. The intensity increasing of some bands corresponding to the formation of polar groups, such as carbonyl and hydroxyl between 1400–1260 cm^−1^, carboxylate or conjugated ketone between 1690–1550 cm^−1^, and aldehyde groups between 1736–1722 cm^−1^ were attributed to the degradation, sustaining the process efficiency, and are in a perfect accordance with the termination phase of reaction mechanisms that were achieved. Morphological investigations through the SEM technique have showed that, after aging, the plasticized starch amount increasing has been reflected in both surface roughness and more micro cracks appearance also. The water uptake and mass loss tests that have been done before and after ageing also have demonstrated that the natural degradation is favored by the addition of plasticized starch in the composite due to the appearance of some strongly hydrophilic groups from natural rubber, plasticized starch, and composite also. In the process of replacing conventional fillers, as silica and carbon black, with some natural fillers in order to obtain composites that are highly degradable in natural environment, starch can be considered as being a solution.

## Figures and Tables

**Table 1:** Percentage modifications of the mechanical properties of unfilled and filled natural rubber (NR) after thermal aging.

Mechanical Property (%)	Sample Type (PS Loading) (phr of PS at 100 phr of NR)					
	NR	NR-10	NR-20	NR-30	NR-40	NR-50
Hardness	−6.67	−4.44	0	0	+1.96	+3.64
Elasticity	−15.38	−16.98	+15.38	+10.00	+10.53	+11.11
100% Modulus	−2.60	+16.67	+36.67	+53.33	+53.33	+65.00
Tensile strength	−14.63	+23.29	−7.61	−8.24	+2.86	+1.96
Elongation at break	+16.33	+33.83	+1.14	+1.22	+24.34	+12.13
Elongation set	0.00	+18.73	+33.33	+23.53	+170.00	+210.00
Tearing strength	+4.55	+1.74	0	+13.33	+20.00	+23.33

**Table 2:** Percentage modifications of the mechanical properties of unfilled and filled NR after one year of natural ageing in temperate climate.

Mechanical Property (%)	Sample Type (PS Loading) (phr of PS at 100 phr of NR)					
	NR	NR-10	NR-20	NR-30	NR-40	NR-50
Hardness	−6.67	−8.89	−2.17	−10.42	−5.88	−9.09
Elasticity	−15.38	−16.98	−15.38	−5.00	−5.26	−5.56
100% Modulus	−94.81	−91.67	−91.67	−91.67	−91.67	−91.67
Tensile strength	−91.64	−86.99	−69.57	−82.35	−96.29	−96.86
Elongation at break	−64.00	−59.20	−67.37	−68.59	−78.50	−76.60
Elongation set	−25.00	−25.00	−26.67	−29.41	+40.00	+60.00
Tearing strength	−40.91	−43.48	−54.55	−36.67	−56.67	−85.33

**Table 3:** Percentage modifications of gel fraction and cross-link densities of unfilled and filled NR after 1 year of natural ageing in temperate climate.

Sample Type (PS Loading)	Gel Fraction		Cross-Link Density	
	Thermal Aging	Natural Aging for 1 Year	Thermal Aging	Natural Aging for 1 Year
NR	+0.47	−0.56	−8.28	−32.13
NR-10	+1.05	−0.83	+0.71	−14.44
NR-20	+1.26	−0.78	−8.07	−37.81
NR-30	+2.24	−3.42	−5.47	−37.83
NR-40	−0.57	−3.55	−2.60	−26.03
NR-50	−0.41	−3.48	−3.99	−28.46

**Table 4:** Characteristic infrared bands observed in NR and NR-PS spectra.

Wave Number (cm^−1^)	Assignment
740–760	C–O–C ring vibration from starch or deformation vibration of R_2_C=CH–R groups from NR
833	=CH out-of-plane bending vibration from NR rubber
870	C(1)–H(α) bending vibration from starch
930–925	skeletal mode vibrations of α-(1-4) glycosidic linkage (C–O–C) from starch
1034–1038	C–O stretching vibration in C–O–H and C–O–C in the anhydrous glucose ring from starch
1080–1086	C–O–C stretching vibration that indicate the grafting of PS on NR
1125–1126	C–O stretching of C–O–C (from starch) or of alcohols >HC–OH resulted from the degradation
1240–1260	carbonyl ((>C=O) and hydroxyl (–OH) compound resulted from the degradation
1310–1315	bending vibration of C–H and C–O groups of aromatic rings (starch)
1370–1380	–CH_3_ asymmetric deformation of NR
1440–1450	–CH_2_– deformation vibration from NR or –CH_2_– symmetric bending vibration from starch
1655–1665	–C=C– stretching vibration in the NR structure or may be due to absorbed water or carboxylate or conjugated ketone (>C=O) resulted from the degradation
1710–1740	the fatty acid ester groups existing in NR or carbonyl group (>C=O) from ketone (R_2_C=O) or aldehyde (RCOH) resulted from the oxidative degradation
2852–2854	–CH_2_– symmetric stretching vibration of NR
2919–2927	–CH_2_– asymmetric stretching vibration of NR
2958–2960	–CH_3_ asymmetric stretching vibration of NR
3030–3040	=CH– stretching vibration of –CH=CH_2_ group from NR
3300–3380	N–H stretching vibration of amide groups from the existing proteins in NR or from OH-stretching vibration (–OH as a result of the degradation by oxidation)

**Table 5:** Mass lose before and after thermal and natural ageing.

Samples Codes	Mass Loss (%)		
	Before Ageing	After Accelerated Ageing	After 1 Year of Natural Ageing
NR	0.256	0.263	0.282
NR-10	0.263	0.286	0.293
NR-20	0.309	0.409	0.417
NR-30	1.059	0.963	1.095
NR-40	2.083	1.031	2.619
NR-50	2.971	1.126	5.960

**Table 6:** The recipes used for composites obtaining.

Ingredients (phr)	Mixtures Codes					
	NR	NR-10	NR-20	NR-30	NR-40	NR-50
Natural rubber (NR)	100	100	100	100	100	100
Starch	0	10	20	30	40	50
Glycerine	0	6	12	18	24	30
Peroxyde	8	8	8	8	8	8
TMPT	3	3	3	3	3	3
Antioxidant	1	1	1	1	1	1

## References

[1] Craciun G., Manaila E., Stelescu M.D. (2016). New Elastomeric Materials Based on Natural Rubber Obtained by Electron Beam Irradiation for Food and Pharmaceutical Use. Materials.

[2] Kruželák J., Sýkora R., Hudec I. (2016). Sulphur and peroxide vulcanisation of rubber compounds-overview. Chem. Pap..

[3] Kruželák J., Sýkora R., Hudec I. (2015). Peroxide vulcanization of natural rubber. Part II: Effect of peroxides and co-agents. J. Polym. Eng..

[4] Henning S.K., Boye W.M. (2009). Fundamentals of Curing Elastomers with Peroxides and Coagents II: Understanding the Relationship Between Coagent and Elastomer. Rubber World.

[5] Rajan R., Varghese S., George K.E. (2013). Role of coagents in peroxide vulcanizatin of natural rubber. Rubber Chem. Technol..

[6] Vieira E.R., Mantovani J.D., de Camargo Forte M.M. (2013). Comparison between peroxide/coagent cross-linking systems and sulfur for producing tire treads from elastomeric compounds. J. Elastom. Plast..

[7] Bucsi A., Szocs F. (2000). Kinetics of radical generation in PVC with dibenzoyl peroxide utilizing high-pressure technique. Macromol. Chem. Phys..

[8] Alvarez Grima M.M. (2007). Novel Co-Agents for Improved Properties in Peroxide Cure of Saturated Elastomers. Ph.D. Thesis.

[9] Drobny J.G. (2012). Ionizing Radiation and Polymers: Principles, Technology and Applications.

[10] Dikland H.G., Ruardy T., Van der Does L., Bantjes A. (1993). New coagents in peroxide vulcanization of EPM. Rubber Chem. Technol..

[11] Thitithammawong A., Uthaipan N., Rungvichaniwat A. (2012). The effect of the ratios of sulfur to peroxide in mixed vulcanization systems on the properties of dynamic vulcanized natural rubber and polypropylene blends. Songklanakarin J. Sci. Technol..

[12] Manaila E., Craciun G., Stelescu M.D., Ighigeanu D., Ficai M. (2014). Radiation vulcanization of natural rubber with polyfunctional monomers. Polym. Bull..

[13] Stelescu M.D., Manaila E., Craciun G., Dumitrascu M. (2014). New Green Polymeric Composites Based on Hemp and Natural Rubber Processed by Electron Beam Irradiation. Sci. World J..

[14] Manaila E., Stelescu M.D., Craciun G., Ighigeanu D. (2016). Wood Sawdust/Natural Rubber Ecocomposites Cross-Linked by Electron Beam Irradiation. Materials.

[15] Datta J. (2015). Effect of Starch Fillers on the Dynamic Mechanical Properties of Rubber Biocomposite Materials. Polym. Compos..

[16] Datta J., Rohn M. (2008). Structure, thermal stability and mechanical properties of polyurethanes, based on glycolysate from polyurethane foam waste prepared with use of 1,6-hexanediol as a glycol. Polimery.

[17] Liu C., Shao Y., Jia D. (2008). Chemically modified starch reinforced natural rubber composites. Polimer.

[18] Mente P., Motaung T.E., Hlangothi S.P. (2016). Natural Rubber and Reclaimed Rubber Composites–A Systematic Review. Polym. Sci..

[19] Lomeli Ramírez M.G., Satyanarayana K.G., Iwakiri S., Bolzon de Muniz G., Tanobe V., Flores-Sahagun T.S. (2011). Study of the properties of biocomposites. Part, I. Cassava starch-green coir fibers from Brazil. Carbohydr. Polym..

[20] Mali S., Grossmann M.V.E., García M.A., Martino M.N., Zaritzky N.E. (2008). Antiplasticizing effect of glycerol and sorbitol on the properties of cassava starch Films. Braz. J. Food Technol..

[21] Gaspar M., Benko Z., Dogossy G., Reczey K., Czigany T. (2005). Reducing water absorption in compostable starch-based plastics. Polym. Degrad. Stab..

[22] Mathew A.P., Dufresne A. (2002). Plasticized waxy maize starch: Effects of polyols and relative humidity on material properties. Biomacromolecules.

[23] Van der Burg M.C., Van der Woude M.E., Janssen L.P.B.M. (1996). The influence of plasticizer on extruded thermoplastics starch. J. Vinyl Addit. Technol..

[24] Ahmed K., Nizami S.S., Raza N.Z., Mahmood K. (2013). Effect of micro-sized marble sludge on physical properties of natural rubber composites. Chem. Ind. Chem. Eng. Q..

[25] Thongsang S., Sombatsompop N. (2007). Dynamic Rebound Behavior of Silica/Natural Rubber Composites: Fly Ash Particles and Precipitated Silica. J. Macromol. Sci. Part B.

[26] Chen R.S., Ahmad S., Ab Ghani M.H., Salleh M.N. (2014). Optimization of High Filler Loading on Tensile Properties of Recycled HDPE/PET Blends Filled with Rice Husk. AIP Conf. Proc..

[27] Nourbakhsh A., Baghlani F.F., Ashori A. (2011). Nano-SiO_2_ filled rice husk/polypropylene composites: Physico-mechanical properties. Ind. Crop. Prod..

[28] Ahmed K. (2015). Hybrid composites prepared from Industrial waste: Mechanical and swelling behavior. J. Adv. Res..

[29] Kukle S., Gravitis J., Putnina A., Stikute A. The effect of steam explosion treatment on technical hemp fibres. Proceedings of the 8th International Scientific and Practical Conference.

[30] Hanafi I., Muniandy K., Othman N. (2012). Fatigue life, morphological studies, and thermal aging of rattan powder-filled natural rubber composites as a function of filler loading and a silane coupling agent. BioResources.

[31] Abdul Kader M., Bhowmick A.K. (2003). Acrylic rubber–fluorocarbon rubber miscible blends: Effect of curatives and fillers on cure, mechanical, aging, and swelling properties. J. Appl. Polym. Sci..

[32] Rattansom N., Prasertsri S. (2009). Relationship among Mechanical properties, heat ageing resistance, cut growth behaviour and morphology in natural rubber: Partial replacement of clay with various type of carbon black at similar hardness level. Polym. Test..

[33] Ismail H., Ishiaku U.S., Azhar A.A., Mohd Ishak Z.A. (1997). A comparative study of the effect of thermo-oxidative aging on the physical properties of rice husk ash and commercial fillers in epoxidized natural rubber compounds. J. Elastom. Plast..

[34] Azura A.R., Ghazali S., Mariatti M. (2008). Effects of the filler loading and aging time on the mechanical and electrical conductivity properties of carbon black filled natural rubber. J. Appl. Polym. Sci..

[35] Khanlari S., Kokabi M. (2010). Thermal stability, aging properties, and flame resistance of NR-based nanocomposite. J. Appl. Polym. Sci..

[36] Martins A.F., Visconte L.L.Y., Schuster R.H., Boller F., Nunes H.C.R., Nunes R.C.R. (2004). Ageing Effect on Dynamic and Mechanical Properties of NR/Cel II Nanocomposites. Kautsch. Gummi Kunstst..

[37] Somers A.E., Bastow T.J., Burgar M.I., Forsyth M., Hill A.J. (2000). Quantifying rubber degradation using NMR. Polym. Degradr. Stab..

[38] Riyajan S.-A., Sasithornsonti Y., Phinyocheep P. (2012). Green natural rubber-g-modified starch for controlling urea release. Carbohydr. Polym..

[39] Fang J.M., Fowler P.A., Tomkinson J., Hill C.A.S. (2002). The preparation and characterization of a series of chemically modified potato starches. Carbohydr. Polym..

[40] Mu T.-H., Zhang M., Raad L., Sun H.-N., Wang C. (2015). Effect of α-Amylase Degradation on Physicochemical Properties of Pre-High Hydrostatic Pressure-Treated Potato Starch. PLoS ONE.

[41] Eng A.H., Tanaka Y., Gan S.N. (1992). FTIR studies on amino groups in purified Hevea rubber. J. Nat. Rubber Res..

[42] Kim I.-S., Lee B.-W., Sohn K.-S., Yoon J., Lee J.-H. (2016). Characterization of the UV Oxidation of Raw Natural Rubber Thin Film Using Image and FT-IR Analysis. Elastom. Compos..

[43] Coates J., Meyers R.A. (2000). Interpretation of Infrared Spectra, A Practical Approach. Encyclopedia of Analytical Chemistry.

[44] Polymer Properties Database. http://polymerdatabase.com/polymer%20chemistry/Thermal%20Degradation.html.

[45] Alberti A., Bertini S., Gastaldi G., Iannaccone N., Macciantelli D., Torri G., Vismara E. (2005). Electron beam irradiated textile cellulose fibres: ESR studies and derivatisation with glycidyl methacrylate (GMA). Eur. Polym. J..

[46] Connors S.A. (1998). Chemical and Physical Characterization of the Degradation of Vulcanized Natural Rubber in the Museum Environment. Master’s Thesis.

[47] Palmas P., Le Campion L., Bourgeisat C., Martel L. (2001). Curing and thermal ageing of elastomers as studied by H-1 Broadband and C-13 high-resolution solid-state NMR. Polymer.

[48] Muniandy K., Hanafi I., Othman N. (2012). Studies on natural weathering of rattan powder filled natural rubber composites. BioResources.

[49] Datta R.N., Bhowmick A.K. (2008). Rubber-curing systems. Current Topics in Elastomers Research.

[50] Noriman N.Z., Ismail H. (2011). The effects of electron beam irradiation on the thermal properties, fatigue life and natural weathering of styrene butadiene rubber/recycledacrylonitrile–butadiene rubber blends. Mater. Des..

[51] Rohana Yahya Y.S., Azura A.R., Ahmad Z. (2011). Effect of Curing Systems on Thermal Degradation Behaviour of Natural Rubber (SMR CV 60). J. Phys. Sci..

[52] Azura A.R., Muhr A.H., Thomas A.G. (2006). Diffusion and reactions of oxygen during ageing for conventionally cured natural rubber vulcanisate. Polym. Plast. Technol. Eng..

[53] Pimolsiriphol V., Saeoui P., Sirisinha C. (2007). Relationship among Thermal Ageing Degradation, Dynamic Properties, Cure Systems, and Antioxidants in Natural Rubber Vulcanisates. Polym. Plast. Technol. Eng..

[54] Hoque M.E., Ye T.J., Yong L.C., Dahlan K.Z.M. (2013). Sago Starch-Mixed Low-Density Polyethylene Biodegradable Polymer: Synthesis and Characterization. J. Mater..

[55] Arroyo M., Lopez-Manchado M.A., Herrero B. (2003). Organo-montmorillonite as substitute of carbon black in natural rubber compounds. Polymer.

[56] Chenal J.M., Chazeau L., Guy L., Bomal Y., Gauthier C. (2007). Molecular weight between physical entanglements in natural rubber: A critical parameter during strain-induced crystallization. Polymer.

